# Myocardial fibrosis in asymptomatic and symptomatic chronic severe primary mitral regurgitation and relationship to tissue characterisation and left ventricular function on cardiovascular magnetic resonance

**DOI:** 10.1186/s12968-020-00674-4

**Published:** 2020-12-14

**Authors:** Boyang Liu, Desley A. H. Neil, Monisha Premchand, Moninder Bhabra, Ramesh Patel, Thomas Barker, Nicolas Nikolaidis, J. Stephen Billing, Thomas A. Treibel, James C. Moon, Arantxa González, James Hodson, Nicola C. Edwards, Richard P. Steeds

**Affiliations:** 1 Department of Cardiology, University Hospital Birmingham, Birmingham, UK; 2 Institute of Cardiovascular Science, University of Birmingham, Birmingham, UK; 3 Department of Cellular Pathology, University Hospital Birmingham, Birmingham, UK; 4 School of Immunology and Immunotherapy, University of Birmingham, Birmingham, UK; 5 Department of Cardiothoracic Surgery, University Hospital Coventry, Coventry, UK; 6 Department of Cardiothoracic Surgery, New Cross Hospital, Wolverhampton, UK; 7 Institute for Cardiovascular Sciences, University College London, London, UK; 8 Department for Cardiac Imaging, Barts Heart Centre, St. Bartholomew’s Hospital, London, UK; 9 Program of Cardiovascular Diseases, CIMA Universidad de Navarra and IdiSNA, Pamplona, Spain; 10 CIBERCV, Instituto de Salud Carlos III, Madrid, Spain; 11 Department of Statistics, Institute of Translational Medicine, Queen Elizabeth Hospital Birmingham, Birmingham, UK; 12 Green Lane Cardiovascular Service, Department of Cardiology, Auckland City Hospital, Auckland, New Zealand; 13 Department of Cardiothoracic Surgery, University Hospital Birmingham, Birmingham, UK

**Keywords:** Mitral regurgitation, Histological fibrosis, Extracellular volume, Late gadolinium enhancement, Myocardial strain, Exercise capacity, Symptom status

## Abstract

**Background:**

Myocardial fibrosis occurs in end-stage heart failure secondary to mitral regurgitation (MR), but it is not known whether this is present before onset of symptoms or myocardial dysfunction. This study aimed to characterise myocardial fibrosis in chronic severe primary MR on histology, compare this to tissue characterisation on cardiovascular magnetic resonance (CMR) imaging, and investigate associations with symptoms, left ventricular (LV) function, and exercise capacity.

**Methods:**

Patients with class I or IIa indications for surgery underwent CMR and cardiopulmonary exercise testing. LV biopsies were taken at surgery and the extent of fibrosis was quantified on histology using collagen volume fraction (CVF_mean_) compared to autopsy controls without cardiac pathology.

**Results:**

120 consecutive patients (64 ± 13 years; 71% male) were recruited; 105 patients underwent MV repair while 15 chose conservative management. LV biopsies were obtained in 86 patients (234 biopsy samples in total). MR patients had more fibrosis compared to 8 autopsy controls (median: 14.6% [interquartile range 7.4–20.3] vs. 3.3% [2.6–6.1], P < 0.001); this difference persisted in the asymptomatic patients (CVF_mean_ 13.6% [6.3–18.8], P < 0.001), but severity of fibrosis was not significantly higher in NYHA II-III symptomatic MR (CVF_mean_ 15.7% [9.9–23.1] (P = 0.083). Fibrosis was patchy across biopsy sites (intraclass correlation 0.23, 95% CI 0.08–0.39, P = 0.001). No significant relationships were identified between CVF_mean_ and CMR tissue characterisation [native T1, extracellular volume (ECV) or late gadolinium enhancement] or measures of LV function [LV ejection fraction (LVEF), global longitudinal strain (GLS)]. Although the range of ECV was small (27.3 ± 3.2%), ECV correlated with multiple measures of LV function (LVEF: Rho = − 0.22, P = 0.029, GLS: Rho = 0.29, P = 0.003), as well as NTproBNP (Rho = 0.54, P < 0.001) and exercise capacity (%PredVO_2_max: R = − 0.22, P = 0.030).

**Conclusions:**

Patients with chronic primary MR have increased fibrosis before the onset of symptoms. Due to the patchy nature of fibrosis, CMR derived ECV may be a better marker of global myocardial status.

*Clinical trial registration* Mitral FINDER study; Clinical Trials NCT02355418, Registered 4 February 2015, https://clinicaltrials.gov/ct2/show/NCT02355418

## Introduction

Chronic primary mitral regurgitation (MR) exposes the left ventricle (LV) to a volume overload, with progressive remodelling, left ventricular (LV) dilatation, and myocardial dysfunction. Despite long-standing guidelines on the indications for surgery in symptomatic and asymptomatic severe chronic primary MR, one fifth of patients continue to present post-operatively with reduced LV ejection fraction (LVEF) and an increased risk of later heart failure [1]. Although there is support for early repair in asymptomatic patients at low risk of complications, there are limited randomised data to separate early repair from watchful waiting. Given the ageing of the population, the proportion of older patients with asymptomatic severe chronic primary MR is growing and these patients do not always find the option even of low risk surgery attractive in the absence of symptomatic benefit. Current class I indications for surgery in asymptomatic individuals require confirmation of severe MR, LV cavity dilatation (end systolic dimension ≥ 4.0 cm) and LVEF ≤ 60%, yet intervention based on these cut-offs is associated with a 50% reduction in long-term survival and 2.5 fold increase in risk of heart failure [2]. Given the limitations of these parameters in the management of primary MR, there is a need for additional information to support either watchful waiting or early surgery [3].

Early autopsy studies in patients with chronic severe primary MR demonstrated a stepwise increase in myocardial fibre hypertrophy and interstitial space with increasing symptoms and LV dysfunction [4]. However, these changes were only found to be significant in those who died with advanced heart failure; meanwhile, animal studies have suggested that progressive development of supra-normal levels of myocardial fibrosis with exhaustion of muscle hypertrophy mechanisms is responsible for the onset of heart failure in MR [5]. While histological data in humans are limited, there has been rapid growth in studies using cardiovascular magnetic resonance (CMR) imaging to characterise LV remodelling and myocardial architecture in primary MR. Late gadolinium enhancement (LGE) has been identified in a significant proportion of patients with chronic severe primary MR before surgery and is thought to reflect focal replacement fibrosis [6, 7]. In previous studies, we and others have demonstrated elevated myocardial T1 relaxation times and expansion of the extracellular volume fraction (ECV), an imaging biomarker of diffuse interstitial fibrosis (DIF), in asymptomatic primary MR [6, 8]. However, histological data in asymptomatic primary MR are lacking, and there are no data linking fibrosis on histology with either T1, ECV, LGE or functional change in the LV. Our hypothesis is that volume overload in severe MR is associated with fibrosis that can be measured by CMR tissue characterisation, and that fibrosis is associated with symptom status, impaired global longitudinal strain and reduced exercise capacity. Therefore, the aims of this prospective study of patients with chronic severe primary MR were to assess: (i) the histological changes within the myocardium; (ii) the association between histological extent of fibrosis and tissue characterisation on CMR; (iii) the relationship between extent of myocardial fibrosis (histology; CMR) on LV function, and exercise capacity.

## Methods

### Study cohort

All patients were enrolled in the prospective multicentre Mitral FINDER study from three tertiary cardiothoracic centres with high volume mitral valve repair programmes (patients undergoing surgery at: Queen Elizabeth Hospital Birmingham n = 52, Coventry Hospital n = 49, New Cross Hospital n = 3; clinicaltrials.gov NCT02355418) between August 2015 and March 2018. Full study inclusion and exclusion criteria have been reported [9], with patients referred for mitral valve surgery based on European Society of Cardiology class I or class IIa indications [10]. In brief, consecutive adult patients aged over 18 years were included, based on the presence of severe primary degenerative MR, diagnosed and quantified on echocardiography according to standard guidelines [11]. Patients were excluded if they had primary MR not due to degenerative disease, secondary MR, congenital heart disease, inherited or acquired cardiomyopathy, a history of myocardial infarction, symptomatic concomitant coronary artery disease, moderate or severe aortic valve disease, pregnancy, or could not undergo either CMR or cardiopulmonary exercise testing (CPET).

All patients underwent clinical assessment with history and examination, blood sampling [including full blood count, haematocrit, renal function, N-terminal pro-brain natriuretic peptide (NTproBNP)], symptom assessment with New York Heart Association (NYHA) classification and multiparametric CMR at the primary study site (Queen Elizabeth Hospital Birmingham).

Classification into fibroelastic deficiency (FED) versus Barlow’s disease was based on independent review of echocardiographic and CMR imaging by two experienced imaging cardiologists (RPS, NCE), and confirmed on surgery.

The study received favourable ethical review from the UK National Research Ethics Service (15/EM/0243) and conformed to the Helsinki Declaration. Subjects gave written consent to participate.

### Histology

During mitral valve surgery, three LV biopsies were obtained from each patient from the LV septum, anterior and posterior free wall, using a 14G TruCut needle or scalpel. Histological analyses were performed blinded to clinical and imaging data (DN, BL). Myocardial connective tissue was analysed on ×20 scanned Masson Trichrome sections (Axio Scan.Z1 ZEISS, Oberkochen, Germany) which stains extracellular connective tissue blue and stains muscle purple-red. Fibrosis burden quantified as collagen volume fraction (CVF) was derived from Ilastik machine-learning based, supervised object classification and segmentation software [12], which was trained in-house against an expert (DN). This system identified and quantified the surface area of connective tissue versus the surface area of muscle. CVF_mean_ represents the mean average of CVF values from the three biopsies originating from an individual patient, and CVF_max_ represents the highest CVF across the three sites for the individual patient, or from all available sites in those where it was not possible to collect all three biopsies.

Control myocardial samples from single whole-heart autopsy sections were obtained from autopsies of eight subjects (five males, all Caucasian, mean age 59 ± 6.9 years). These data allowed comparison of age-related histological changes in subjects who died of non-cardiac causes with no evidence of macroscopic or microscopic cardiac lesions.

Measurement of cardiomyocyte hypertrophy was performed by manually quantifying the cross-sectional area (CSA) of 50 randomly selected cardiomyocytes via ZEN software (Carl Zeiss Microscopy). To ensure consistency, only cardiomyocytes found in its mid-axial orientation (denoted by presence of central nuclei) were eligible for quantification.

### Cardiovascular magnetic resonance

LV and right ventricular (RV) volumes and mass, and left atrial volumes were acquired in line with 1.5T CMR scanner (Avanto; Siemens Healthineers, Erlangen, Germany) protocols [13, 14]. Single breath hold modified Look-Locker inversion recovery sequence (MOLLI) was used for T1 mapping in the LV base and mid-ventricular short axis levels before and between 15 and 20 min after contrast administration (3, 3, 5 scheme), according to previously published parameters [8]. LGE imaging was performed 7 to 10 min after 0.15 mmol/kg of gadolinium based-contrast agent (Gadovist Bayer Healthcare, Berlin, Germany).

CMR imaging analyses were performed offline using Cvi42^®^ (version 5.3.6, Circle Cardiovascular Imaging, Calgary, Canada) by an operator (BL) blinded to all demographic and descriptive data, and without information regarding any clinical parameters. For ventricular volume analysis, the endocardial border was detected with thresholding, delineation of atria/ventricles was confirmed in matched long axis planes, and papillary muscles and trabeculations were included in ventricular mass. For T1 mapping, two short axis maps (base and mid LV) were manually contoured for endo- and epicardial borders. Partial voluming of blood was minimised by using a 20% offset from the endo- and epicardial border [15]. ECV values stated in the manuscript are global values. Blood was taken for measurement of haematocrit at the same time as the CMR scans. Additional region-of-interest (ROI) ECV values were obtained from the mid LV septum, anterior free wall and posterior free wall, to mirror the origins of histological biopsy collection. Myocardial deformation on CMR was quantified using feature tracking of cine images with Cvi42^®^, as per previously described methodology [16, 17]. LGE mass was quantified automatically using a signal intensity thresholding of > 3 standard deviations above reference mean [18].

### Symptom status and exercise testing

Symptom status was assessed using the NYHA classification status, as assessed by the responsible clinician, with NYHA I classified as asymptomatic and NYHA II–IV as symptomatic. Objective testing of exercise capacity was carried out using maximal exertion treadmill CPET, with incremental ramp protocols as per American Thoracic Society guidelines [19].

### Statistical analyses

The baseline demographics of the cohort were summarized, with continuous variables reported as means ± standard deviations (SDs) where normally distributed, and as medians and interquartile ranges (IQRs) otherwise. Comparisons of continuous variables between groups were performed using independent samples t-tests for normally distributed variables, or Mann–Whitney U tests otherwise, with chi^2^-tests used to analyze nominal variables. Associations between continuous variables were assessed using Spearman’s (rho) or Pearson’s (R) correlation coefficients. Where applicable, these associations were further quantified using linear regression models. The goodness of fit of these regression models was assessed graphically, with logarithmic transformations applied where either poor fit or heteroscedasticity were identified. All statistical analyses were performed using SPSS (version 24, Statistical Package for the Social Sciences, International Business Machines, Inc., Armonk, New York, USA), and P < 0.05 was deemed to be statistically significant throughout.

## Results

In total, 120 consecutive patients with severe primary degenerative MR were recruited, of whom 105 were referred for mitral valve repair. One patient referred for surgery was subsequently excluded due to a requirement for percutaneous coronary intervention, leaving n = 104 for analysis, of whom 39 were referred for a class I indication (symptoms) and 65 were referred for a class IIa indication (high likelihood of repair). Only one patient required MV replacement. As expected given the indications for surgery, most of the cohort were asymptomatic, with 63% (n = 65) New York Heart Association (NYHA) class 1, 30% (n = 31) NYHA class II, and 8% (n = 8) NYHA class III. Demographic, histology and imaging data are summarised in Table 1. All 15 patients that declined surgery were asymptomatic with a class IIa indication for repair, but chose to be treated conservatively. Patients choosing conservative management were significantly older with lower estimated glomerular filtration rate (eGFR), but had smaller LV end-systolic volume index (LVESVI) and less MR (Additional file 1: Table S1).

**Table 1:** Baseline characteristics of surgical patients according to symptom status

	N	Asymptomatic N = 65	Symptomatic N = 39	P-value
Clinical characteristics				
Age (years)	104	62 ± 14	64 ± 12	0.428
Male sex	104	50 (77%)	26 (67%)	0.254
BMI (kg/m^2^)	104	25 ± 4	27 ± 5	0.065
BSA (m^2^)	104	1.88 ± 0.23	1.91 ± 0.23	0.491
Treated hypertension	104	20 (31%)	12 (31%)	1.000
Atrial fibrillation	104			*0.008*
None		53 (82%)	21 (54%)	
Paroxysmal		2 (3%)	5 (13%)	
Permanent		10 (15%)	13 (33%)	
eGFR (ml/min/1.73m^2^)	104	75 (65–84)	72 (60–82)	0.401
Degeneration subtype	104			0.076
Barlow’s disease		24 (37%)	11 (28%)	
Indeterminant		3 (5%)	7 (18%)	
Fibroelastic deficiency		38 (58%)	21 (54%)	
MLHFQ score	103	5 (1–17)	43 (30–57)	*< 0.001*
%PredVO_2_max (%)	103	99 ± 20	79 ± 22	*< 0.001*
Resting PASP (mmHg)	85	33 ± 10	38 ± 20	0.071
Ventricular ectopy burden (%)	54	0.1 (0.0–1.0)	0.5 (0.0–1.4)	0.356
Cardiac magnetic resonance characteristics				
LVEDVI (ml/m^2^)	104	105 ± 22	103 ± 21	0.663
LVESVI (ml/m^2^)	104	33 ± 13	35 ± 11	0.342
LVESD (mm)	104	3.7 ± 0.7	3.8 ± 0.6	0.380
LVMI (g/m^2^)	104	71 ± 13	66 ± 13	*0.045*
LVEF (%)	104	70 ± 8	66 ± 9	0.065
GCS (%)	102	− 18.2 ± 3.0	− 16.6 ± 3.5	*0.017*
GLS (%)	102	− 15.9 ± 2.7	− 14.8 ± 3.3	0.099
LAVI (ml/m^2^)	102	88 ± 44	96 ± 47	0.341
RVESVI (ml/m^2^)	104	31 ± 9	33 ± 13	0.474
RVEF (%)	104	57 ± 8	55 ± 10	0.122
RV E_ll_	104	− 21.6 ± 4.5	− 21.4 ± 5.0	0.811
MR volume (ml)	104	62 ± 27	67 ± 36	0.410
MR fraction (%)	104	44 ± 12	49 ± 19	0.121
ECV (%)	101	26.8 ± 3.1	28.2 ± 3.3	*0.045*
Native T1 (ms)	103	981 ± 24	991 ± 26	0.054
LGE presence (n, %)	102	22 (34%)	12 (32%)	0.830
LGE quantification (g)	102	0.00 (0.00–0.37)	0.00 (0.00–0.24)	0.452

Data are reported as N (%), with P-values from chi^2^ tests; median (interquartile range), with P-values from Mann–Whitney U tests; or as mean ± SD, with P-values from independent samples t-tests, as applicable. Italic P-values are significant at P < 0.05

*%PredVO*_*2*_*max* percentage predicted maximal oxygen consumption, *BMI* body mass index, *ECV* extracellular volume, *eGFR* estimated glomerular filtration rate, *GCS* global circumferential strain, *GLS* global longitudinal strain, *LGE* late gadolinium enhancement, *LAVI* left atrial volume indexed, *LVEDVI* left ventricular diastolic volume indexed, *LVEF* left ventricular ejection fraction, *LVESD* left ventricular end-systolic dimension, *LVESVI* left ventricular systolic volume indexed, *LVMI* left ventricular mass indexed, *MLHFQ* Minnesota living with heart failure questionnaire, *MR* mitral regurgitation, *RVEF* right ventricular ejection fraction, *RV E*_*ll*_ right ventricular longitudinal strain, *RVESVI* right ventricular end-systolic volume indexed, *PASP* pulmonary artery systolic pressure

### Histology of myocardial biopsies in severe chronic primary MR

Of the 104 patients undergoing MV repair, 234 biopsies were collected from 86 patients. Biopsies were taken from all three LV sites (septum, anterior and posterior free wall) in n = 67, with n = 14 having two biopsies, and n = 5 a single biopsy. Biopsies were not feasible across all biopsy sites due to limited visualisation or safe instrument access to the ventricular myocardium from the left atrial incision at the time of surgery. In addition, two patients chose to undergo surgery at institutions external to the study sites, where biopsies were not taken.

#### Myocardial fibrosis

**Fig. 1:**
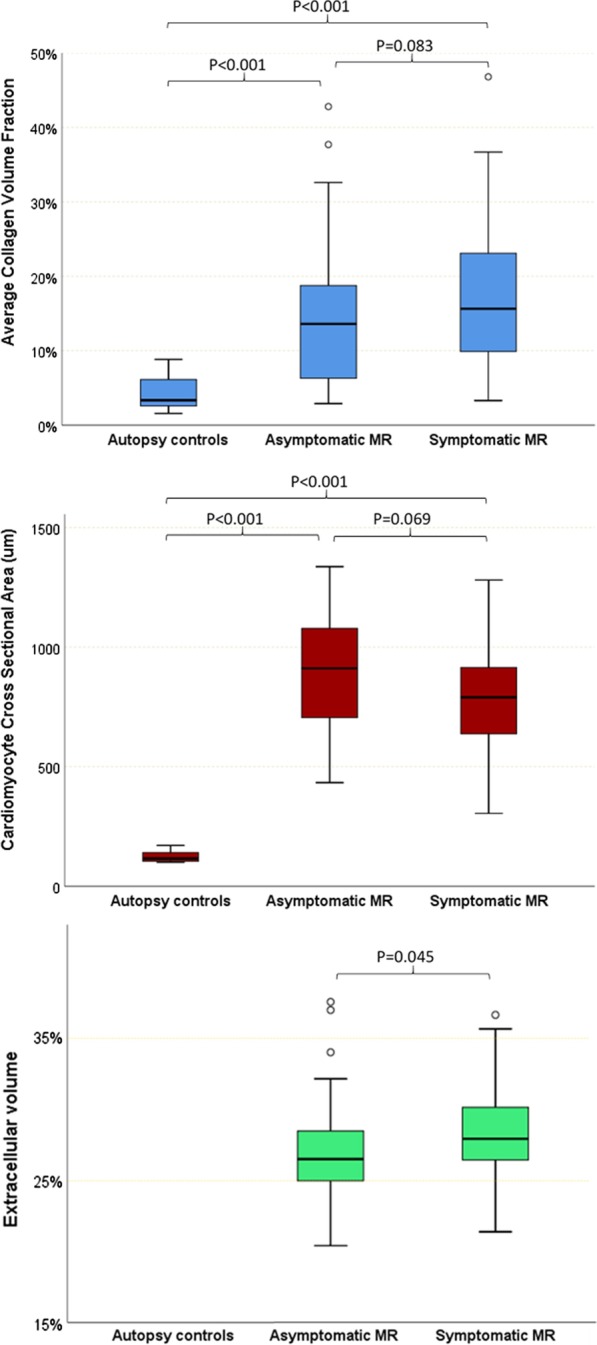
Boxplots illustrating the distribution of mean collagen volume fraction (CVF_mean_) (top) and cardiomyocyte cross sectional area (CSA) (bottom) in autopsy controls vs. asymptomatic and symptomatic mitral regurgitation (MR) patients. P values are derived from Mann–Whitney U tests for non-parametric variables (collagen volume fraction) and independent T-test for parametric variables (cardiomyocyte cross sectional area, extracellular volume). *MR* mitral regurgitation

MR patients had a median CVF_mean_ of 14.6% [IQR 7.4–20.3] and CVF_max_ of 22.2% [10.2–31.8], which were significantly higher than control CVF values (3.3% [2.6–6.1], P < 0.001). The difference in fibrosis persisted when limited to asymptomatic MR patients compared to controls (CVF_mean_ 13.6% [6.3–18.8], P < 0.001). Although there was a trend towards increased fibrosis in symptomatic patients compared to asymptomatic MR patients based on NYHA classification, this was not significant (CVF_mean_ 15.7% [9.9–23.1] (P = 0.083) (Fig. 1; results remain non-significant when taking biopsy location and exclusion/inclusion of endocardium into account, Additional file 1: Table S3). There were no gender-related differences in fibrosis (CVF_mean_ male 14.1% [7.3–18.5] vs. female 16.1% [11.4–24.6], P = 0.163).

**Fig. 2:**
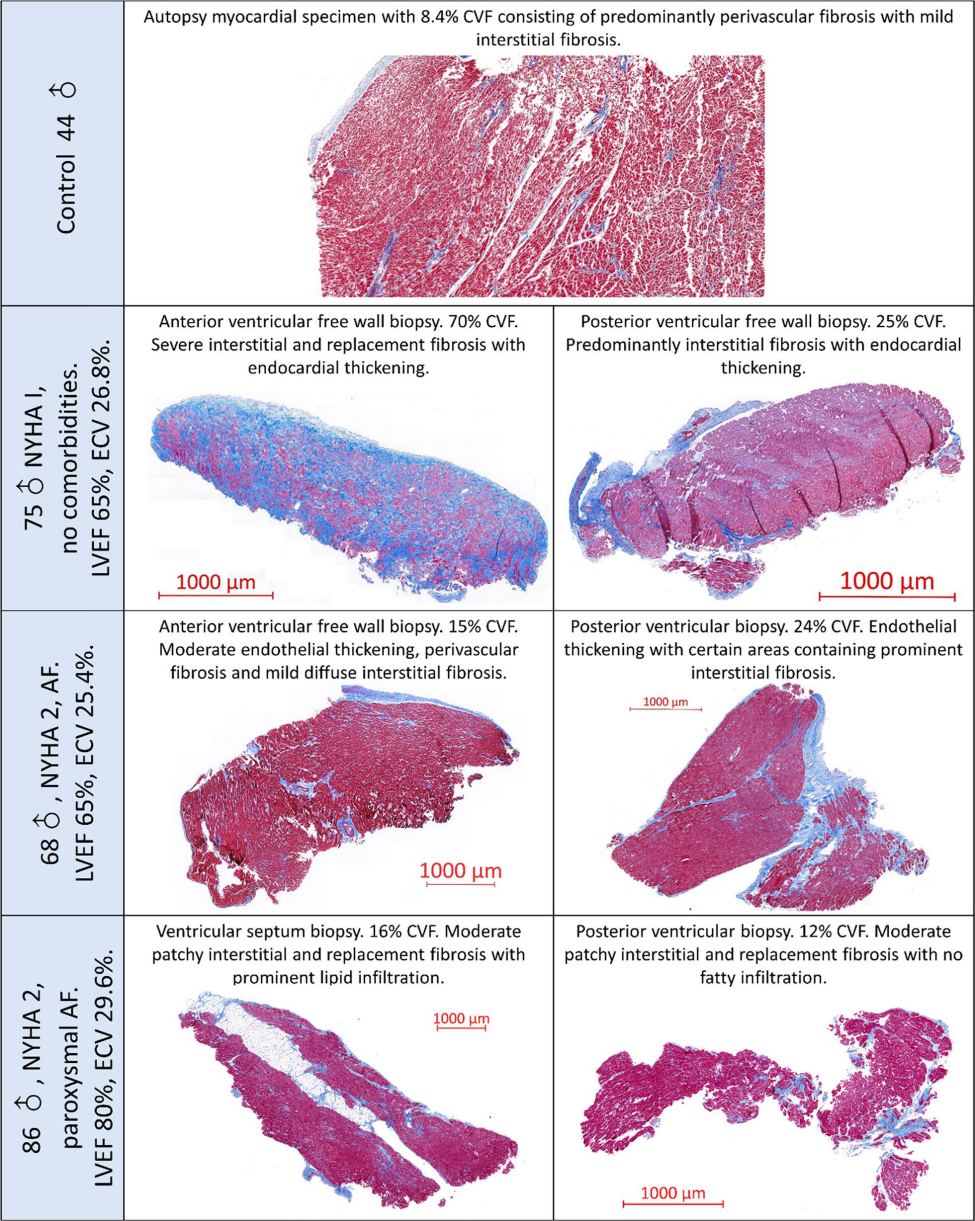
Histological specimens from mitral regurgitation patients and controls. Sections are stained with Masson Trichrome highlighting fibrous tissue as blue and myocytes as purple-red. Histological fibrosis is patchy with little congruence in fibrosis burden across different biopsy sites. Extracellular volume fraction (ECV) can provide an overall estimation of myocardial status, but fails to account for endocardial fibrosis

A total of 124 myocardial biopsies contained endocardium (median thickness 100 µm [58–181]), and 111 biospies contained only myocardium. Biopsies with endocardium showed higher CVF than those without endocardium (16.7% [10.4–27.1] vs. 6.6% [3.5–12.3], P < 0.001). There was a significant correlation between mean endocardial thickness and CVF (rho = 0.35, P < 0.001), consistent with endocardial thickening and subendocardial scar. The pattern of fibrosis varied between MR patients and within the myocardium, including diffuse interstitial fibrosis, perivascular fibrosis and areas of replacement fibrosis (Fig. 2).

There was no clear pattern to the distribution of myocardial fibrosis within each individual biopsy sample; although CVF in samples with endocardium was approximately twice that of mid-myocardial samples, no clear endocardial–epicardial gradient could be seen within the myocardium of each biopsy sample. In the 67 patients who had three biopsies, this heterogeneity was reflected in a low intraclass correlation coefficient (ICC) for fibrosis across the three biopsy sites (ICC = 0.23, 95% CI 0.08–0.39, P = 0.001).

#### Cellular hypertrophy

Direct histological quantification of cardiomyocyte hypertrophy was performed via haematoxylin and eosin (H&E) stained slides. MR patients were found to possess marked cardiomyocyte hypertrophy compared to controls (CSA 853 ± 230 µm^2^ vs 124 ± 26 µm^2^, P < 0.001, Fig. 1) with histological quantification correlating significantly with CMR derived LV mass indexed (LVMI; R = 0.36, P = 0.001). There was a trend for cardiomyocyte CSA in symptomatic patients to be lower than asymptomatic patients (799 ± 218 µm^2^ vs 891 ± 232 µm^2^, P = 0.069).

#### Fatty infiltration

During examination of surgical biopsies, fat infiltration between cardiomyocytes was noted. As a potential confounder to T1 and ECV measurements (T2 mapping was not performed), fat infiltration was semi-quantitatively assessed by an experienced cardiac histopathologist blinded to clinical and imaging data using a scale of 0 (no fat infiltration) to 3 (prominent fat infiltration). Of 79 biopsies assessed, 52 (66%) contained no fat infiltration, 15 (19%) mild; 10 (13%) moderate; and 2 (3%) prominent fat infiltration (Fig. 2). Although extent of fat infiltration increased significantly with CVF_mean_ (rho = 0.23, P = 0.045), there were no signficiant correlations with CMR parameters, including native T1 or ECV. There was however, a near-significant correlation with age (rho = 0.22, P = 0.056).

### Histology and CMR measurement of myocardial fibrosis

**Fig. 3:**
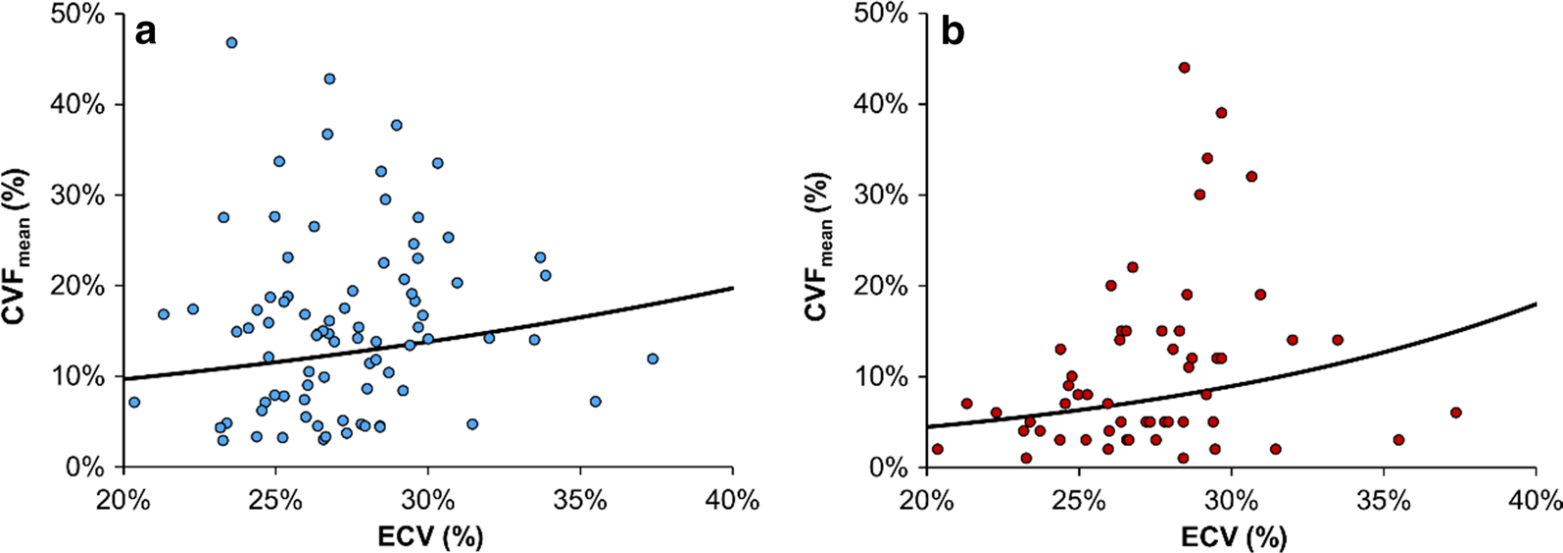
Relationship between ECV and histological evidence of interstitial fibrosis on biopsy (CVFmean) for **a** all biopsies, and **b** biopsies containing only myocardium. The trendlines are based on regression models, with ECV as the independent variable, and Log_2_CVFmean as the independent variable. This relationship was not found to be statistically significant when all biopsies were included within analyses (**a**, Rho = 0.18, P = 0.101, N = 83), but became statistically significant when limiting analyses to biopsies that contained only myocardium (**b**, Rho = 0.33, P = 0.015, N = 56)

The extent of histological fibrosis quantified on biopsy by CVF_mean_ did not correlate significantly with either with native T1 (rho = 0.04, P = 0.714), or ECV (rho = 0.18, P = 0.101) regardless of whether analyses were performed according to global values (Fig. 3a) or according to region of interest drawn to match biopsy site (Additional file 2: Figure S1). However, a statistically significant association between CVF_mean_ and ECV was present when eliminating endocardium from any analysis and limiting histological quantification to myocardium only (n = 56, rho = 0.33, P = 0.015; Fig. 3b). Conversely, no association was present between ECV and CVF_mean_ quantified from biopsies containing endocardium (n = 64, rho = 0.03, P = 0.822).

LGE was present in 34 (33%) of 102 patients who received gadolinium-based contrast agent, with a median mass of 0.98 g (IQR 0.33–2.47). Mid-myocardial LGE was located in the basal inferolateral LV segment (n = 23), and/or in the papillary muscles (n = 8), basal anterolateral segment (n = 3), mid inferolateral segment (n = 2) and mid septum (n = 1). The extent of histological fibrosis quantified on biopsy by CVF_mean_ was not significantly correlated with the amount of left ventricular LGE (rho = − 0.09, P = 0.402).

### Myocardial fibrosis, LV function and exercise capacity

The extent of histological fibrosis quantified on biopsy by CVF_mean_ was not significantly correlated with CMR measured LV volumes, LVEF or LV mass, and there was no relationship with other imaging markers of LV function, including GLS and E/eʹ. There was however, a signficant positive correlation between CVF_mean_ and NTproBNP (Rho = 0.35, P = 0.001) (Table 2). Sub-analysis using CVF_mean_ derived from biopsy samples without endocardium did not result in any alterations in statistical significance (data not shown). There was a consistent and significant correlation however, between ECV and multiple parameters of LV function (LVEF, LVESVI, global longitudinal strain (GLS) and E/eʹ), as well as NTproBNP. The amount of LGE in the cohort was small and there was no significant difference in CMR measured LVEF, LVESVI or GLS, E/eʹ or NTproBNP between patients with or without LGE.

**Table 2:** Correlation of histological fibrosis against non-invasive pre-operative imaging parameters

	Correlation with CVF_mean_			Correlation with ECV		
	N	Rho	P-value	N	Rho	P-value
LVEDVI (ml/m^2^)	86	− 0.02	0.853	101	0.12	0.234
LVESVI (ml/m^2^)	86	0.07	0.541	101	0.22	*0.025*
LVMI (g/m^2^)	86	− 0.12	0.261	101	− 0.05	0.640
LVEF (%)	86	− 0.15	0.178	101	− 0.22	*0.029*
GCS (%)^b^	84	0.14	0.221	99	0.31	*0.002*
GLS (%)^b^	84	0.12	0.271	99	0.29	*0.003*
LAVI (ml/m^2^)	84	0.17	0.123	99	0.26	*0.008*
Echocardiography E/eʹ	72	0.09	0.460	87	0.25	*0.022*
NTproBNP	84	0.35	*0.001*	99	0.54	*< 0.001*
RVESVI (ml/m^2^)	86	− 0.10	0.381	101	0.21	*0.037*
RVEF (%)	86	− 0.06	0.579	101	− 0.14	0.170
RV E_ll_	86	0.09	0.411	101	0.30	*0.003*
MR volume (ml)	86	0.04	0.714	101	− 0.07	0.471
MR fraction (%)	86	0.16	0.147	101	0.02	0.868
ECV (%)	83	0.18	0.101	–	–	–
Native T1 (ms)	85	0.04	0.714	101	0.62	*< 0.001*
LGE presence^a^	84	− 0.07	0.514	101	0.06	0.535
PASP (mmHg)	71	0.14	0.232	82	0.14	0.209
Degeneration subtype (Barlow’s)^a^	77	0.21	0.070	91	0.13	0.208

Results are from Spearman’s (Rho) correlation coefficients. Italic P-values are significant at P < 0.05

*ECV* extracellular volume, *GCS* global circumferential strain, *GLS* global longitudinal strain, *LAVI* left atrial volume indexed, *LGE* late gadolinium enhancement, *LVEDVI* left ventricular diastolic volume indexed, *LVEF* left ventricular ejection fraction, *LVESVI* left ventricular systolic volume indexed, *LVMI* left ventricular mass indexed, *MR* mitral regurgitation, *NTproBNP* N-terminal pro-brain natriuretic peptide, *PASP* pulmonary artery systolic pressure, *RVEF* right ventricular ejection fraction, *RV E*_*ll*_ right ventricular longitudinal strain, *RVESVI* right ventricular end-systolic volume indexed

^a^Dichotomous variables

^b^GCS and GLS are expressed as negative values, therefore a positive correlation between ECV and strain suggests that increased ECV is related with worse (less negative) strain

Four patients undergoing surgery were unable to complete cardiorespiratory symptom-limited CPET due to non-cardiorespiratory causes (severe osteoarthritis and anxiety). Mean peak oxygen consumption (VO_2_) was 23.2 ± 7.4 ml/kg/min, giving a %PredVO_2_max of 92.9 ± 20.5%, at an age-predicted heart rate of 99.0 ± 14.8%. On linear regression, formally tested exercise capacity quantified as %PredVO_2_ max was significantly associated with ECV, markers of biventricular systolic function, E/eʹ, the presence of atrial fibrillation, and pulmonary artery systolic pressure, but not with CVF_mean_ (Table 3).

**Table 3:** Linear regression analyses of exercise capacity against histology, cardiac imaging and pulmonary pressures

	Relationship with %PredVO_2_max			
	N	R	Coefficient (95% CI)	P-value
Age (per year)	100	0.05	0.08 (− 0.22, 0.38)	0.598
Gender (female)^b^	100	− 0.01	− 0.31 (− 9.43, 8.80)	0.946
BMI (kg/m^2^)	100	− 0.01	− 0.03 (− 1.01, 0.95)	0.945
Treated hypertension (yes)^b^	100	0.04	1.75 (− 7.09, 10.59)	0.695
Permanent AF (yes)^b^	100	− 0.32	− 15.60 (− 24.97, − 6.23)	*0.001*
Log_2_CVF_mean_ (%)^a^	83	− 0.18	− 3.82 (− 8.45, 0.82)	0.105
Log_2_CVF_max_ (%)^a^	83	− 0.11	− 2.20 (− 6.42, 2.03)	0.304
Cardiomyocyte CSA (um^2^)	83	0.13	0.01 (− 0.01, 0.03)	0.245
Echocardiography E/eʹ	87	− 0.36	− 1.60 (− 2.49, − 0.70)	*0.001*
PASP (mmHg)	81	− 0.28	− 0.43 (− 0.76, − 0.10)	*0.012*
Degeneration subtype (Barlow’s)^b^	91	0.02	0.77 (− 8.18, 9.72)	0.865
LVEF (%)	100	0.23	0.57 (0.09, 1.05)	*0.021*
GCS (%)	98	− 0.26	− 1.64 (− 2.87, − 0.42)	*0.009*
GLS (%)	98	− 0.32	− 2.19 (− 3.50, − 0.87)	*0.001*
LVESVI (ml/m^2^)	100	− 0.20	− 0.34 (-0.67, − 0.01)	*0.045*
LVMI (g/m^2^)	100	0.03	0.05 (− 0.26, 0.36)	0.739
RVEF (%)	100	0.41	0.96 (0.54, 1.39)	*< 0.001*
RV E_ll_	100	− 0.20	− 0.84 (− 1.69, 0.01)	0.051
RVESVI (ml/m^2^)	100	− 0.23	− 0.44 (− 0.83, − 0.06)	*0.024*
MR volume (ml)	100	− 0.19	− 0.13 (− 0.26, 0.00)	0.057
MR fraction (%)	100	− 0.40	− 0.55 (− 0.80, − 0.30)	*< 0.001*
ECV (%)	98	− 0.22	− 1.37 (− 2.60, − 0.13)	*0.030*
Native T1 (ms)	100	− 0.07	− 0.06 (− 0.22, 0.10)	0.471
LGE presence (yes)^b^	99	0.01	0.33 (− 8.42, 9.08)	0.941

Results are from Pearson’s (R) correlation coefficients and univariable regression models with %PredVO_2_max as the dependent variable. Italic P-values are significant at P < 0.05

*AF* atrial fibrillation, *BMI* body mass index, *CSA* cross-sectional area, *CVF* collagen volume fraction, *ECV* extracellular volume, *GCS* global circumferential strain, *GLS* global longitudinal strain, *LGE* late gadolinium enhancement, *LVEF* left ventricular ejection fraction, *LVESVI* left ventricular systolic volume indexed, *LVMI* left ventricular mass indexed, *MR* mitral regurgitation, *RVEF* right ventricular ejection fraction, *RV E*_*ll*_ right ventricular longitudinal strain, *RVESVI* right ventricular end-systolic volume indexed, *PASP* pulmonary artery systolic pressure

^a^Factors were log_2_-transformed to improve model fit, hence coefficients are relative to a two-fold increase in the factor

^b^Factors are dichotomous; hence the coefficient represents the difference in the outcome between the stated category and the reference category

### Impact of MR subtype

Sub-group analysis of patients with BD demonstrated higher CVF_max_ compared to FED patients (median 28.0% [IQR 16.7–42.8] vs. 20.2% [7.5–29.4], P = 0.009), but not CVF_mean_ (15.4% [12.1–25.3] vs. 14.0% [5.5–19.1], P = 0.071). There were no significant differences in biventricular volumes, LVEF, LVMI or MR severity; ECV and LGE prevalence were also similar (Additional file 1: Table S2).

## Discussion

This is the most comprehensive study to characterise myocardial changes in patients with chronic primary severe MR, combining CMR based assessment of structural changes with detailed invasive histological profiling, and the identification of functional correlates.

Our work provides definitive histological evidence for the presence of myocardial fibrosis, even in asymptomatic MR patients with normal range CMR volumetric parameters and good exercise capacity. We noted complex morphology and topography of fibrosis with three main patterns: thickened endocardium with increased fibrosis in biopsies containing sub endocardium; cellular hypertrophy and increased fibrosis compared to controls; and a variable degree of fat infiltration. Despite the increase in fibrosis compared to control subjects without MR, both in asymptomatic and symptomatic patients, the extent of fibrosis on myocardial biopsy at the time of surgery was not related to LVEF, GLS, diastolic function (E/e′; left atrial volume) or MR severity. Biopsy and CMR offered complementary information however, because patterns of fibrosis proved variable, and CMR-derived ECV and LGE were not able to capture the subendocardial changes highlighted on histology. Hence, while the extent of fibrosis on myocardial biopsy was related to symptoms but was not related to LV function, non-invasive tissue characterisation with ECV was associated with changes in both systolic and diastolic function, and measured exercise capacity.

The new finding of significant myocardial fibrosis in asymptomatic patients contrasts with early autopsy data, which found a significant increase in the size of the interstitial space only in those patients with severe MR (NYHA Class III–IV) who died early after mitral valve replacement from cardiac failure with low cardiac output syndrome [4]. Although the study did document an increase in size of the interstitial space in less symptomatic patients with severe MR (NYHA Class II–III) but who died early after mitral valve replacement from causes other than cardiac failure, this was not significant compared to autopsy controls. We believe there are technical issues and issues of sensitivity that explain these differences. Firstly, the retrospective study by Fuster et al. used hearts collected in 10% formalin over a 10-year period 1962–1972 and quantified the interstitial space using photographs on which the interstitial space was highlighted in white pen, without histological characterisation. In contrast, samples in our study were stained for fibrosis that was then quantified directly, as was the degree of fatty infiltration. Secondly, we suspect that ECV may be a more sensitive 3D measure of the interstitial space, while acknowledging that the range of ECV observed within our surgical cohort (27.3 ± 3.2%) was only mildly raised compared to the mean ECV of 25.9% (95% CI 25.5–26.3%) within a recent pooled analysis of 3872 participants [20]. This low-grade ECV expansion observed within our study may be related to disease duration, which is difficult to quantify, but most of our patients were under follow-up in valve clinic and were referred for early surgery, while LV size, function and mass were normal, and with unrestricted exercise capacity on formal testing. This latter pooled analysis also highlights the large confidence intervals and significant overlap in T1 and ECV values between health and disease [20], which reflects the recognised limitations of ECV and T1 analyses.

Despite evidence that high native T1, elevated ECV, and LGE measure diffuse interstitial and replacement fibrosis in other diseases [21–24], we found no correlation between overall CVF_mean_ and CMR measures of fibrosis. There are a number of potential reasons for this. Similar to previous reports [7], we noted the incidence of LGE to be 34% in our MR cohort, but the most common location was in the basal inferolateral LV wall and associated papillary muscles. Although the inferolateral wall was one of the three pre-specified targets to be biopsied, the focal nature of the fibrosis means this could have been missed. This situation is best exemplified by the low sensitivity of myocardial biopsy in the diagnoses of cardiac sarcoidosis [25], and mirrors the data for aortic valve diseases where some groups have been able to demonstrate a significant correlation between histological and CMR quantified fibrosis [24], whilst others have not [26]. We report a low ICC for CVF from multiple biopsy sites within the same heart; this finding is reminiscent of the large (43%) coefficient of variation reported for interstitial fibrosis (but only 3% for cardiomyocyte CSA) in dilated cardiomyopathy [27]. This may also help to explain why ECV—a measure of global myocardial status, correlated with CMR measures of biventricular systolic function and echo derived E/e, highlighting the potential value of ECV as a marker of LV remodelling in MR. Finally, avoidance of the endocardium during quantification of ECV to prevent blood pool contamination may also contribute to the discordant results with histology, a hypothesis which is supported by our sub-analyses on myocardium-only derived CVF. However, ECV in itself has limitations, including cross over of ranges between health and disease, practical limitations of movement with respiration, and further research is needed on whether ECV offers incremental prognostic value over traditional cardiac biomarkers such as NTproBNP.

## Limitations

This study was observational in nature and therefore no causation can be inferred. Although pre-specified, invasive myocardial biopsy was not performed in all patients with symptomatic and asymptomatic MR who went forward to surgery due to technical difficulties. Despite this, we provide the largest histologic characterisation of patients with severe primary MR. We were unable to anatomically match the location of the autopsy sections with the location of our histological biopsies. However, there is no evidence for significant regional variations in CVF quantity in the healthy heart and therefore this limitation should not detract from the significant difference in CVF observed between MR patients and controls. Similarly, anatomical matching of the location of biopsy collection with the commonly observed location LGE in MR is difficult, and is likely to contribute towards the lack of association between CVF and LGE quantification. Invasive measurement of pulmonary artery pressures was performed at the clinician’s discretion and was not a requirement of this study. Although assessment by echocardiography was performed, there were cases inevitably without enough tricuspid regurgitation for pulmonary artery systolic pressure to be measured. Finally, the narrower range of global fibrosis quantified by ECV may have weakened our ability to detect correlations, where a broader range always favours the likelihood of obtaining significant results. However, studying a group of severe MR patients early in their disease process increases the clinical relevance of our findings given the trend for earlier surgical assessment of asymptomatic patients.

## Conclusions

Histological evidence of diffuse interstitial and replacement myocardial fibrosis, along with concomitant cardiomyocyte hypertrophy is present in asymptomatic MR, in the presence of normal range LV volume and LVEF. Fibrosis on biopsy is patchy and highly variable within the heart, which may contribute to the lack of correlation with T1 mapping, ECV and LGE. ECV, as a marker of global myocardial status, correlated well with multiple measures of ventricular function in MR. Further studies are warranted to investigate whether latent fibrosis detected using T1, ECV or LGE offer incremental value in predicting post-operative outcome in asymptomatic patients with severe primary MR.

## Supplementary information

**Additional file 1: Table S1. **Clinical characteristics of conservative and surgical patients. **Table S2. **Cardiac magnetic resonance parameters according to subtype of MR.** Table S3. **CVF_mean_ values according to symptom status and biopsy type.

**Additional file 2: Figure S1**. Scatter plots demonstrating the correlation between regional ECV and CVF in the A) septum (rho = − 0.05, P = 0.683), B) anterior wall (rho = − 0.02, P = 0.886) and C) posterior wall (rho = 0.22, P = 0.150) of the left ventricle.

## Data Availability

The datasets used and/or analyzed supporting the conclusions of the article are available from the corresponding author on reasonable request.

## Abbreviations

BMI: Body mass index
BSA: Body surface area
CMR: Cardiovascular magnetic resonance
CPET: Cardiopulmonary exercise testing
CSA: Cross sectional area
CVF_mean_: Mean collagen volume fraction
CVF_max_: Maximum collagen volume fraction
DIF: Diffuse interstitial fibrosis
ECV: Extracellular volume fraction
eGFR: Estimated glomerular filtration rate
FED: Fibroelastic deficiency
GCS: Global circumferential strain
GLS: Global longitudinal strain
LAVI: Left atrial volume index
LGE: Late gadolinium enhancement
LV: Left ventricle/left ventricular
LVEDVI: Left ventricular end-diastolic volume indexed to body surface area
LVEF: Left ventricular ejection fraction
LVESD: Left ventricular end-systolic diameter
LVESVI: Left ventricular end-systolic volume indexed to body surface area
LVMI: Left ventricular mass indexed to body surface area
MOLLI: Modified Look-Locker inversion recovery
MR: Mitral regurgitation
NTproBNP: N-terminal pro-brain natriuretic peptide
NYHA: New York Heart Association
PASP: Pulmonary artery systolic pressure
ROI: Region of interest
RV: Right ventricle/right ventricular
RVEF: Right ventricular ejection fractino
VO_2_: Oxygen consumption
