# Supplementary material for: Myocardial fibrosis in asymptomatic and symptomatic chronic severe primary mitral regurgitation and relationship to tissue characterisation and left ventricular function on cardiovascular magnetic resonance
Source: J Cardiovasc Magn Reson. 2020 Dec 14;22:86. doi: 10.1186/s12968-020-00674-4 (PMC7734760; doi:10.1186/s12968-020-00674-4)
Supplement: Supplementary file 1 — Additional file 1: Table S1. Clinical characteristics of conservative and surgical patients. Table S2. Cardiac magnetic resonance parameters according to subtype of MR. Table S3. CVFmean values according to symptom status and biopsy type. [file 12968_2020_674_MOESM1_ESM.docx]

**Table S1** Clinical characteristics of conservative and surgical patients.

|  |  | **Management** | |  |
| --- | --- | --- | --- | --- |
|  | **N** | ***Conservative*** | ***Surgical*** | **P-value** |
| ***Clinical characteristics*** | | | | |
| Age (years) | 119 | 74±9 | 63±13 | **0.004** |
| Male sex (n, %) | 119 | 8 (53%) | 76 (73%) | 0.206 |
| Treated hypertension | 119 | 8 (53%) | 32 (31%) | 0.084 |
| BMI (kg/m^2^) | 119 | 24±3 | 26±5 | 0.170 |
| Atrial fibrillation | 119 |  |  | 0.227 |
| *None* |  | 9 (60%) | 74 (71%) |  |
| *Paroxysmal (n, %)* |  | 0 (0%) | 7 (7%) |  |
| *Permanent (n, %)* |  | 6 (40%) | 23 (22%) |  |
| eGFR (ml/min/1.73m^2^) | 117 | 56 (43–79) | 75 (64–84) | **0.023** |
| Degeneration subtype | 119 |  |  | 0.347 |
| *Barlow’s* |  | 6 (40%) | 35 (34%) |  |
| *Indeterminant* |  | 3 (20%) | 10 (10%) |  |
| *Fibroelastic deficiency* |  | 6 (40%) | 59 (57%) |  |
| NTproBNP | 117 | 423 (169-1159) | 233 (110-567) | 0.100 |
| MLHFQ score | 119 | 8 (0-21) | 13 (4-41) | 0.201 |
| %PredVO_2_max (%) | 118 | 98±19 | 91±22 | 0.253 |
| Echocardiography E/e’ | 102 | 9.2 (6.2-11.1) | 8.0 (6.9-11.8) | 0.861 |
| Resting sPAP (mmHg) | 99 | 41±16 | 35±15 | 0.184 |
| ***Cardiac magnetic resonance characteristics*** | | | | |
| LVEDVi (ml/m^2^) | 119 | 104.2±21.7 | 86.3±23.6 | **0.004** |
| LVESVi (ml/m^2^) | 119 | 25.6±9.9 | 33.3±11.9 | **0.018** |
| LVEF (%) | 119 | 70.5±7.4 | 68.3±8.2 | 0.324 |
| GCS | 117 | -17.9±3.3 | -17.6±3.2 | 0.752 |
| GLS | 117 | -16.0±4.6 | -15.5±3.0 | 0.542 |
| LVMi (g/m^2^) | 119 | 61.2±13.2 | 69.4±13.3 | **0.028** |
| LAVi (ml/m^2^) | 117 | 90.8±44.8 | 76.5±30.5 | 0.236 |
| RVESVi (ml/m^2^) | 119 | 28.5±9.5 | 31.6±10.3 | 0.285 |
| RVEF (%) | 119 | 57.5±8.8 | 56.3±8.8 | 0.599 |
| RV E_ll_ | 118 | -20.8±5.9 | -21.6±4.7 | 0.561 |
| Aortic forward flow (ml) | 115 | 66.9±21.4 | 70.9±20.3 | 0.512 |
| MR volume (ml) | 119 | 40.3±16.8 | 63.9±30.5 | **0.004** |
| MR fraction (%) | 119 | 36.4±11.6 | 46.0±14.8 | **0.017** |
| ECV (%) | 115 | 27.7±2.1 | 27.3±3.2 | 0.687 |
| Native T1 (ms) | 117 | 995.7±29.3 | 985.0±24.9 | 0.143 |
| LGE presence (n,%) | 117 | 5 (33%) | 34 (33%) | 1.000 |
| LGE quantification (g) | 117 | 0.00 (0.00-1.60) | 0.00 (0.00-0.35) | 0.631 |

*Data are reported as N (%), with p-values from chi^2^ tests; median (interquartile range), with p-values from Mann-Whitney U tests; or as mean±SD, with p-values from independent samples t-tests, as applicable. Bold p-values are significant at p<0.05.*

*Abbreviations: %PredVO_2_max percentage predicted maximal oxygen consumption, ECV extracellular volume, eGFR estimated glomerular filtration rate, GCS global circumferential strain, GLS global longitudinal strain, LGE late gadolinium enhancement, LVEF left ventricular ejection fraction, LVESVi left ventricular systolic volume indexed, LVMi left ventricular mass indexed, MLHFQ Minnesota living with heart failure questionnaire, MR mitral regurgitation, NTproBNP N-terminal pro-brain natriuretic peptide, RVEF right ventricular ejection fraction, RV E_ll_ right ventricular longitudinal strain, RVESVi right ventricular end-systolic volume indexed, sPAP systolic pulmonary artery pressure.*

**Table S2** Cardiac magnetic resonance parameters according to subtype of MR.

|  | **Fibroelastic**  **deficiency**  **N=59** | **Barlow’s**  **disease**  **N=35** | **P-value** |
| --- | --- | --- | --- |
| LVESVi (ml/m^2^) | 33.4±11.3 | 32.8±13.6 | 0.833 |
| LVEF (%) | 68.2±8.6 | 69.3±8.0 | 0.554 |
| LVMi (g/m^2^) | 71.4±12.9 | 67.8±13.8 | 0.213 |
| RVESVi (ml/m^2^) | 30.7±9.7 | 33.0±10.5 | 0.294 |
| RVEF (%) | 57.0±9.0 | 55.1±8.0 | 0.316 |
| Aortic forward flow (ml) | 73.9±22.2 | 68.0±17.4 | 0.182 |
| MR volume (ml) | 65.5±31.6 | 65.3±29.6 | 0.975 |
| MR fraction (%) | 45.9±15.2 | 47.5±14.2 | 0.614 |
| LGE presence (n,%) | 21 (37%) | 11 (31%) | 0.597 |
| ECV (%) | 27.0±3.4 | 27.8±3.1 | 0.276 |
| Native T1 (ms) | 981.2±23.0 | 991.6±25.4 | **0.045** |

*Data are reported as mean±SD, with p-values from independent samples t-tests, or as N (%), with p-values from chi^2^-tests, as applicable. Bold p-values are significant at p<0.05.*

*Abbreviations: ECV extracellular volume, LGE late gadolinium enhancement, LVEF left ventricular ejection fraction, LVESVi left ventricular systolic volume indexed, LVMi left ventricular mass indexed, MR mitral regurgitation, RVEF right ventricular ejection fraction, RVESVi right ventricular end-systolic volume indexed.*

**Table S3** CVF_mean_ values according to symptom status and biopsy type

|  | **Asymptomatic** | **Symptomatic** | **P-Value** |
| --- | --- | --- | --- |
| CVF_mean_ from biopsies without endocardium | 6 (4-15)  N=38 | 11 (4-14)  N=20 | 0.480 |
| CVF_mean_ from biopsies with endocardium | 18 (7-24)  N=39 | 17 (13-28)  N=28 | 0.309 |

*Data are reported as median (interquartile range), with p-values from Mann-Whitney U tests.*
