# Supplementary material for: Myocardial fibrosis in asymptomatic and symptomatic chronic severe primary mitral regurgitation and relationship to tissue characterisation and left ventricular function on cardiovascular magnetic resonance
Source: J Cardiovasc Magn Reson. 2020 Dec 14;22:86. doi: 10.1186/s12968-020-00674-4 (PMC7734760; doi:10.1186/s12968-020-00674-4)
Supplement: Supplementary file 2 — Additional file 2: Figure S1. Scatter plots demonstrating the correlation between regional ECV and CVF in the A) septum (rho = − 0.05, P = 0.683), B) anterior wall (rho = − 0.02, P = 0.886) and C) posterior wall (rho = 0.22, P = 0.150) of the left ventricle. [file 12968_2020_674_MOESM2_ESM.docx]

**Figure S1**. Scatter plots demonstrating the correlation between regional ECV and CVF in the A) septum (rho= -0.05, P=0.683), B) anterior wall (rho= -0.02, P=0.886) and C) posterior wall (rho= 0.22, P=0.150) of the left ventricle.


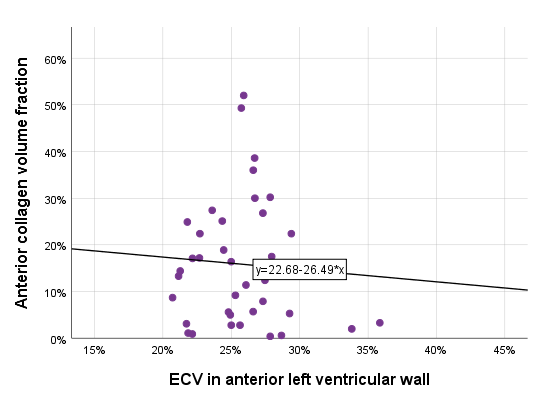

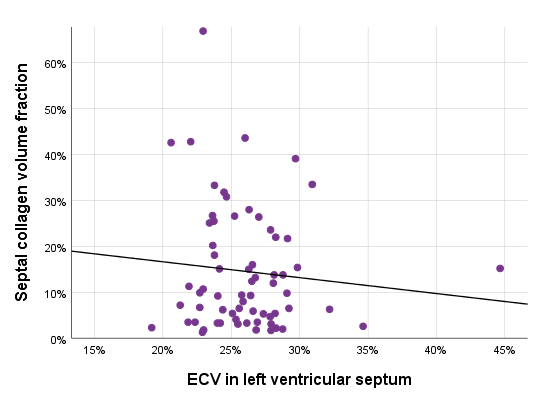

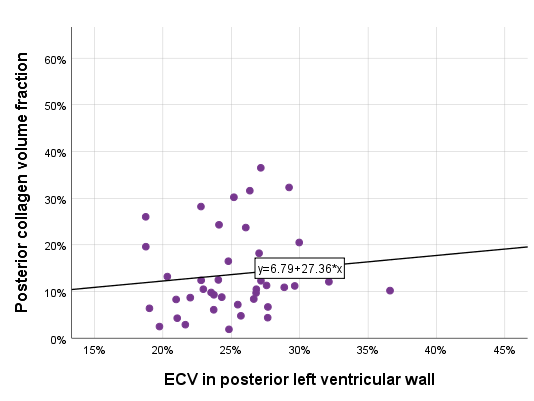


**A**

**B**

**C**
